# Serum IL-10 from systemic lupus erythematosus patients suppresses the differentiation and function of monocyte-derived dendritic cells

**DOI:** 10.7555/JBR.26.20120115

**Published:** 2012-10-31

**Authors:** Zhida Sun, Rong Zhang, Huijuan Wang, Pengtao Jiang, Jiangquan Zhang, Mingshun Zhang, Lei Gu, Xiaofan Yang, Miaojia Zhang, Xiaohui Ji

**Affiliations:** a Department of Oral Mucosal Diseases, the Affiliated Stomatological Hospital, Nanjing Medical University, Nanjing, Jiangsu 210029, China;; b Laboratory Center for Basic Medical Sciences, Nanjing Medical University, Nanjing, Jiangsu 210029, China;; c Department of Microbiology and Immunology, Nanjing Medical University, Nanjing, Jiangsu 210029, China;; d Department of Rheumatology, the First Affiliated Hospital, Nanjing Medical University, Nanjing, Jiangsu 210029, China.

**Keywords:** lupus erythematosus systemic (SLE), interleukin-10 (IL-10), dendritic cells (DCs), differentiation

## Abstract

The role played by cytokines, other than interferon (IFN)-α, in the differentiation and function of dendritic cells (DCs) in systemic lupus erythematosus (SLE), remains unclear. Serum interleukin-10 (IL-10) levels are generally elevated in SLE patients, which might modulate the differentiation of DCs. In this study, DCs were induced from monocytes either by transendothelial trafficking or by culture with granulocyte-macrophage colony-stimulating factor (GM-CSF) + IL-4 + tumor necrosis factor (TNF)-α. Both systems were used to investigate the effects of elevated serum IL-10 level on DC differentiation in SLE patients. The results showed that monocyte-derived DCs induced by either SLE serum or exogenous IL-10 reduced the expression of human leukocyte antigen (HLA)-DR and CD80, decreased IL-12p40 level, and increased IL-10 level, and exhibited an impaired capacity to stimulate allogenic T-cell proliferation. These results indicate that serum IL-10 may be involved in the pathogenesis of SLE by modulating the differentiation and function of DCs.

## INTRODUCTION

Dendritic cells (DCs) are key regulators in immune responses capable of priming naive T cells and initiating primary T-cell responses when they are pulsed with antigenic peptides or proteins, and they are also capable of inducing anergy in autoreactive T cells. T-cell populations show functional disorders in systemic lupus erythematosus (SLE), such as abnormal activation of autoreactive T cells, and defects in responses to microbial antigens. These disorders may result from underlying defects in the function of DCs. It is indicated that both DCs and cytokines are involved in the induction of autoimmune diseases[1]. Phenotypic and functional abnormalities have been described in DCs isolated from patients with SLE[2] and it is believed that abnormal DCs play an important role in the pathogenesis of SLE. It has been shown that interferon (IFN)-α derived from the sera of SLE patients induces normal monocytes to differentiate into DCs, which could capture antigens from apoptotic cells and present them to CD4^+^ T cells[3]. The central role of DCs and IFN-α in SLE has received much attention[4]-[6]. Recently, the IFN-induced protein, IFN-induced protein with tetratricopeptide repeats 4 (IFIT4), was found to be associated with SLE, and to promote the differentiation of monocytes into DC-like cells, supporting the key role of IFN-α in the pathogenesis of SLE[7]. However, there is a disorder of multiple cytokines in patients with SLE, in whom abnormal elevation of interleukin (IL)-1β, IL-6, IL-10, IL-17 or IL-23 is detected[8]-[10]. Our previous study demonstrated that IL-6 in SLE serum significantly affected the differentiation and function of DCs derived from CD34^+^ haematopoietic precursor cells (HPCs) and promoted the expression of human leukocyte antigen (HLA)-DR, CD80 and CD86, IL-10 production and the ability to stimulate allogenic T-cell proliferation, while decreasing IL-12 secretion by DCs[11]. The role played by cytokines, other than IFN-α and IL-6, in the differentiation and function of DCs in SLE patients remains unclear till now.

IL-10 has both immunosuppressive and immunostimulatory properties, and its potential for dampening inflammatory responses has attracted much interest in research of autoimmune diseases[12]. Increased IL-10 production by SLE peripheral blood B cells and monocytes is observed, which is thought to correlate with disease activity[13]. Inhibition of IL-10 activity by neutralizing antibodies results in decreased expression of disease markers in both SLE patients and murine models of lupus[14],[15]. The results of these studies are explained by the intrinsically high levels of IL-10 related to lupus susceptibility and severity through the promotion of B-cell proliferation, and immune stimulation by this cytokine seems to trump immunesuppression in lupus patients. However, the effect of serum IL-10 on the differentiation and function of DCs in pathogenesis of SLE remains unclear. IL-10 has been demonstrated to inhibit the differentiation and function of DCs by reducing the expression of co-stimulators and major histocompatibility complex (MHC)-II molecule and IL-12 production[16]-[18]. It is, therefore, hypothesized that increased levels of IL-10 in the serum of SLE patients may modulate the differentiation of DCs. The major purpose of this study was to obtain the direct evidence of the effects of IL-10 present in the serum of SLE patients on the differentiation and function of monocyte-derived DCs (MDDCs).

## SUBJECTS AND METHODS

### Subjects

Totally 50 SLE patients recruited during routine clinic visits to the Department of Rheumatology, the First Affiliated Hospital of Nanjing Medical University, were enrolled in this study. All of the patients met the revised SLE criteria of the American Rheumatism Association[19]. The patients were all female, with a median age of 29 years (range, 24-37 years). Thirteen age-matched healthy female volunteers served as controls. Informed consent was obtained from all subjects.

### Serum preparation

The blood samples were collected from all participants and allowed to clot at room temperature for 2 h. The sera were separated by centrifugation at 2000 rpm for 10 min and stored in aliquots at -20°C until required. The IL-10 concentrations in the sera from SLE patients were measured using ELISA reagent kits (Diaclone Research; Besancon Cedex, France) according to the manufacturer's instructions. The sera with different levels of IL-10 were selected, grouped and pooled, and the sera were grouped as follows: Group 1, SLE sera with normal levels of IL-10 (< 10 pg/mL); Group 2, SLE sera with mildly elevated levels of IL-10 (10-20 pg/mL); Group 3, SLE sera with highly elevated levels of IL-10 (20-40 pg/mL).

### Separation of peripheral blood mononuclear cells (PBMCs)

Peripheral blood from healthy donors was supplied by Jiangsu Province Blood Centre. Heparinized blood (200 mL) was collected from each donor and PBMCs were isolated by standard Ficoll-Hypaque density-gradient centrifugation for 2 h. PBMCs were then washed twice with phosphate buffered saline (PBS) before use.

### Induction of DCs

DCs were induced from monocytes by two methods, transendothelial trafficking and culture with granulocyte-macrophage colony-stimulating factor (GM-CSF) + IL-4 + tumor necrosis factor α (TNF-α). Transendothelial trafficking was performed using a method modified from Randolph et al.[20]. Fresh human umbilical cord was collected from Nanjing Maternity and Children Health Care Hospital. Within 4 h of delivery the umbilical vein was washed with PBS and the endothelial layer was digested with 0.25% trypsin (Invitrogen; Carlsbad, CA, USA). Human umbilical vein endothelial cells (HUVECs) were collected and identified by factor VIII-related antigen staining using fluorescence microscopy (Olympus; Tokyo, Japan). HUVECs were grown on polymerized collagen gels and cultured in RPMI 1640 (GIBCO; Carlsbad, CA, USA) supplemented with 20% fetal calf serum (FCS) (Hyclone; Waltham, MA, USA) for 2 d until full confluency. PBMCs from healthy donors were then incubated with the endothelial monolayer for 2 h before a thorough and careful wash to remove non-migrated cells. The phenotypes (CD14, CD11c, HLA-DR, CD80, and CD86) of both the freshly isolated PBMCs before incubation, and the non-migrated cells after incubation, were analyzed by flow cytometry to enumerate the migrated population. After a further culture for 36 h, the cells that had reverse-transmigrated across the endothelial monolayer were harvested and identified.

In the GM-CSF+IL-4+TNF-α culture system, monocytes were isolated from PBMCs by adhesion to plastic plates for 1 h followed by culture in RPMI 1640 containing 20% FCS in the presence of 1,000 U/mL GM-CSF and 500 U/mL IL-4 (PeproTech; Rocky Hill, NJ, USA). Half of the culture medium was exchanged with fresh medium and cytokines on d 3 and 5, and 1,000 U/mL TNF-α (PeproTech; Rocky Hill, NJ, USA) was added on d 5. Cells were harvested on d 8.

To observe the effects of IL-10 on the differentiation of DCs, normal serum, SLE serum, SLE serum plus anti-IL-10 neutralizing antibodies, SLE serum plus rabbit anti-human IgG isotype controls or normal serum supplemented with exogenous rhIL-10 were added into the medium, respectively. The human serum concentration was 10%. Neutralizing antibodies, isotype controls or exogenous cytokines purchased from PeproTech (Rocky Hill, NJ, USA) were added into the serum at 4°C 24 h prior to use. The optimal neutralizing dose of anti-IL-10 polyclonal antibodies was determined in preliminary dose-response experiments (3-15 ng/mL).

### Morphological observation

DCs induced by the transendothelial trafficking method were collected and observed by light microscopy and scanning electron microscopy (SEM). Cells were washed twice with PBS, transferred onto slides and spun. After fixing in methanol, cells were stained with Wright-Giemsa solution and analyzed under a light microscope (Olympus; Tokyo, Japan; original magnification, ×100). DCs were fixed using 2.5% glutaraldehyde for SEM and post-fixed in 1% osmium tetroxide. Following dehydration through ethyl alcohol and propylene oxide, the cells were critical point-dried in CO_2_ and sputter-coated with gold. Samples were then analyzed under a scanning electron microscope (JEOL; Tokyo, Japan; original magnification, ×8000).

### Phenotypic analysis

Cells were washed, re-suspended in PBS and then conjugated with anti-CD14-fluorescein isothiocyanate (FITC), anti-CD11c-FITC, anti-HLA-DR-phycoerythrin (PE), anti-CD80-PE, anti-CD86-PE, anti-CD83-PE, anti-CD1a-FITC monoclonal fluorescent antibodies or isotype controls (eBioscience; San Diego, CA, USA) at 4°C for 30 min. After washing twice with PBS, cells were analyzed using flow cytometry (BD Biosciences; San Jose, CA, USA). Data analysis was performed using the software CellQuest (BD Biosciences).

### Determination of cytokine levels produced by DCs

After washing twice with PBS, cells were seeded at a concentration of 1.5×10^5^/mL onto 24-well plates and incubated in RPMI 1640 supplemented with 20% FCS for 48 h. The concentrations of IL-12p40, IFN-γ and IL-10 in the culture supernatants were measured using commercial ELISA reagent kits (Diaclone Research; Besancon Cedex, France) according to the manufacturer's instructions

### Measurement of allostimulatory capability of DCs

The allostimulatory capability of DCs was measured in an allogeneic mixed leukocyte reaction (MLR). CD3^+^ T cells purified from PBMCs by a magnetic cell sorting system using anti-human CD3 microbeads (Miltenyi Biotec; Bergisch Gladbach, Germany) were used as effector cells, and were seeded at a density of 2×10^5^ cells/well onto 96-well microtiter plates (Corning; Lowell, MA, USA). The harvested DCs were used as stimulator cells and seeded at DC:T cell ratios of 1:10, 1:20, 1:50 and 1:100. The cells were co-incubated in RPMI 1640 supplemented with 10% FCS for 96h. T cell proliferation was then measured by tritiated thymidine ([^3^H] TdR) incorporation or by cell counting kit-8 (CCK-8, Dojindo Laboratories; Kumamoto, Japan)[21]. For measurement using the [^3^H] TdR incorporation method, 0.5 µCi/well [^3^H] TdR (Amersham; Braunschweig, Germany) was added for the last 16 h of co-culture. [^3^H] TdR incorporation was then measured using a β-scintillation counter (Perkin Elmer; Shelton, CT, USA). Results were expressed as the stimulation index (SI) calculated using the following formula. SI = (CPM_experimental well_-CP_Mblank well_)/ (CP_Mnegative control_ - CP_Mblank well_), while CPM indicates cell counts per min. For detection using CCK-8, the cells were pulsed with 20 µL/well CCK-8 solution and incubated for 2 h. The optical density (OD) was then measured at 450 nm using a microplate reader (CliniBio; Eugendorf, Austria). Results were expressed as SI calculated using the following formula. SI=(OD_experimental well_-OD_blank well_)/(OD_negative control_-OD_blank well_). Each experiment was done in triplicate.

### Statistical analysis

All data were presented as mean±standard deviation (SD), and all statistical analyses were performed using the statistical software SPSS version 11.5 (SPSS Inc; Chicago, IL, USA). Two-group paired comparisons were assessed using paired t-test, and one-way analysis of variance (ANOVA) was used to test the difference of means among groups. The Student-Newman-Keuls (SNK) method was used for pairwise comparisons of the means. A *P*-value < 0.05 was considered statistically significant.

## RESULTS

### SLE serum affects the differentiation and function of DCs induced by transendothelial trafficking

**Fig. 1:**
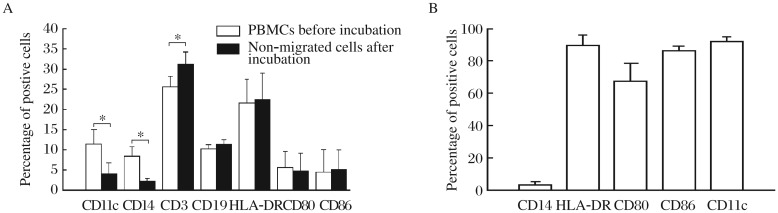
Effects of SLE serum on differentation of PBMCs. A: Phenotypic comparison between PBMCs and non-migrated cells before and after incubation with the monolayer of human umbilical vein endothelial cells. PBMCs are incubated with the endothelial monolayer for 2 h before a thorough and careful wash to remove any non-migrated cells. Expression of CD11c, CD14, CD3, CD19, HLA-DR, CD80 and CD86 by the PBMCs before incubation, and by the non-migrated cells after incubation, are determined by flow cytometry. Results are expressed as mean±SD of percentage of the positive cells. The data are obtained from six independent experiments. *P < 0.05. B: Phenotypic analysis of the reverse-transmigrated cells cultured in the transendothelial. Expression of CD14, HLA-DR, CD80, CD86 and CD11c on the cell surface determined by flow cytometry. Cells are conjugated with fluorescent monoclonal antibodies. Re-sults of six separate experiments are expressed as mean±SD.

HUVECs were identified by factor VIII-related antigen staining. Intensive green fluorescence was observed in the cytoplasm of most HUVECs by fluorescence microscopy (***supplementary Fig. 1A*** available online). After culture on polymerized collagen gels for 2 d, HUVECs formed confluent endothelial monolayers (***supplementary Fig. 1B*** available online). PBMCs were incubated on these monolayers for 2 h and then the non-migrated cells were removed. As shown in ***Fig. 1A***, the proportion of CD11c^+^ and CD14^+^ cells in the non-migrated cells significantly decreased after the incubation, while the number of CD3^+^ cells increased compared with that seen in the PBMCs before the incubation. No significant changes in the proportion of HLA-DR^+^, CD80^+^, CD86^+^ and CD19^+^ cells were observed, indicating that monocytes (CD11c^+^ CD14^+^), rather than T (CD3^+^) or B cells (CD19^+^), migrated through the endothelial monolayer.

**Fig. 2:**
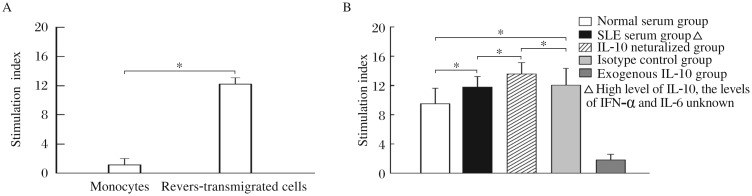
Effects of SLE serum on functions of PBMCs. A: Allostimulatory capacity of the reverse-transmigrated cells induced by the transendothelial trafficking model. Either the reverse-transmigrated cells induced by the transendothelial trafficking model, or the monocytes isolated from PBMCs, are used as stimulator cells and incubated with allogeneic CD3^+^ T cells at a ratio of 1:10. T-cell proliferation is measured by the [^3^H] TdR incorporation method. The degree of proliferation is indicated as the stimulation index (SI). Results are expressed as mean±SD. Data are obtained from six independent experiments. * *P* < 0.05. B: Effect of SLE serum on the allostimulatory capacity of MDDCs induced by the transendothelial trafficking model. MDDCs induced by the transendothelial trafficking model while culturing with different sera are used as stimulator cells and allogeneic CD3+ T cells are used as effec-tor cells. The two kinds of cells are incubated with each other at a ratio of 1:10. T-cell proliferation is measured by the [3H] TdR incorporation method. The degree of proliferation is indicated as the stimulation index (SI). Normal serum group, DCs are induced by the normal serum; SLE serum group, DCs are induced by the SLE serum in which IL-10 level is highly elevated (20-40 pg/mL) while the levels of other cytokines are not measured; IL-10 neutralized group, DCs are induced by the SLE serum plus anti-IL-10 neutralizing antibodies; Isotype control group, DCs are induced by the SLE serum plus rabbit anti-human IgG isotype controls; Exogenous IL-10 group, DCs are induced by the normal serum supplemented with exogenous IL-10 (30 pg/mL). Results are expressed as mean±SD. Data are obtained from six independent experiments. * *P* < 0.05.

After further culture for 36 h, the cells that reverse-transmigrated across the endothelial monolayer were harvested and identified. Under a light microscope and scanning electron microscope, these cells displayed typical morphology of DCs, with many dendritic-like protrusions on the cell surface (***supplementary Fig. 2*** available online). These cells expressed low level of CD14 and high level of HLA-DR, CD80, CD86 and CD11c (***Fig. 1B***), and showed a strong capacity to stimulate allogeneic T cell proliferation (***Fig. 2A***). According to these characteristics, the reverse-transmigrated cells induced by the transendothelial trafficking model were considered as DCs.

In the transendothelial trafficking model, MDDCs induced by the SLE serum (in which IL-10 level was elevated while the levels of other cytokines were not measured), displayed a significantly increased capacity to stimulate allogenic T cell proliferation compared with those induced by the normal serum. Neutralization of IL-10 in the SLE serum led to further increases in the stimulatory capacity of the MDDCs. In contrast, MDDCs induced by the normal serum supplemented with exogenous IL-10 showed a significantly decreased allostimulatory capability than those induced by the normal serum alone (***Fig. 2B***). These results suggest that, in SLE serum, some factors such as IFN-α and IL-6, rather than IL-10, enhance the capacity of MDDCs to stimulate allogenic T cell proliferation, while IL-10 inhibits this capacity.

**Fig. 3:**
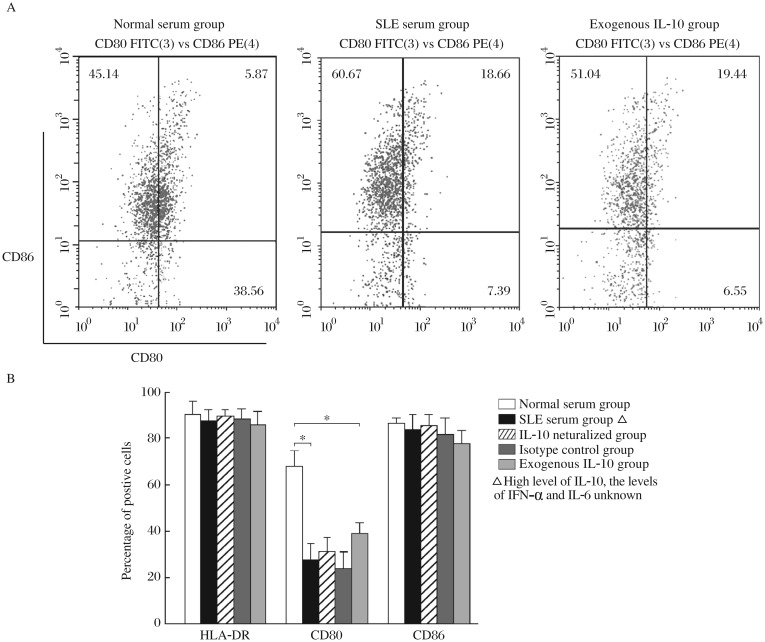
Effects of SLE serum on the phenotype of MDDCs induced by the transendothelial trafficking model. MDDCs are induced by the transendothelial trafficking model and the expression of HLA-DR, CD80 and CD86 is determined by flow cytometry. Normal serum group, MDDCs are induced by the normal serum; SLE serum group, MDDCs are induced by the SLE serum with highly elevated levels of IL-10 (20-40 pg/mL) and normal levels of ILα and IL-6; IL-10 neutralized group, MDDCs are induced by the SLE serum plus anti-IL-10 neutralizing antibodies; Isotype control group, MDDCs are induced by the SLE serum plus rabbit anti-human IgG isotype controls; Exogenous IL-10 group, MDDCs are induced by the normal serum supplemented with exogenous IL-10 (30 pg/mL). A: The results of flow cytometry of different groups. B: The statistical results of flow cytometry. Results are expressed as mean±SD of the percentage of positive cells. Data are obtained from six independent experiments. *P< 0.05.

Therefore, the SLE serum containing highly elevated levels of IL-10 and normal levels of IFN-α and IL-6 was used in the following experiments. MDDCs induced by the SLE serum containing highly elevated levels of IL-10 and normal levels of IFN-α and IL-6, or MDDCs induced by the normal serum supplemented with exogenous IL-10, led to lower percentage of CD80-expressing cells compared with those induced by the normal serum alone. However, the percentages of HLA-DR-and CD86-expressing cells were not changed (***Fig. 3***).

### IL-10 in SLE serum decreases the expression of HLA-DR and CD80 by MDDCs induced by the GM-CSF + IL-4 + TNF-α culture system

**Fig. 4:**
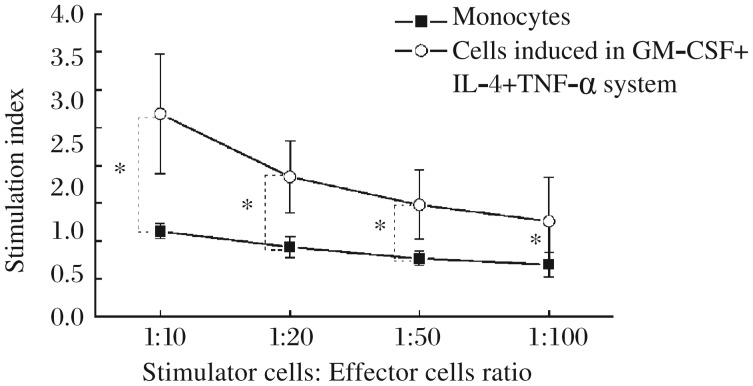
Allostimulatory capacity of the cells induced by the GM-CSF + IL-4 + TNF-α culture system. The cells induced by GM-CSF + IL-4 + TNF-α, or monocytes isolated from PBMCs, are used as stimulator cells and incubated with allogeneic CD3^+^ T cells at ratios of 1:10, 1:20, 1:50 and 1:100. T-cell proliferation is measured using cell counting kit-8. The degree of proliferation is indicated as the stimulation index (SI). Results are expressed as mean±SD. Data are obtained from five independent experiments. * *P* < 0.05.

Monocytes were isolated from PBMCs and cultured with GM-CSF + IL-4 + TNF-α. After 8 d of induction, these cells expressed high levels of HLA-DR, CD80 and CD86 (86.86%±6.92%, 86.62%±7.40% and 93.65%±4.34%, respectively, *n* = 5), and CD83 and CD1a (32.63%±9.49% and 43.11%±6.49%, respectively, *n* = 5). These cells also showed a strong capacity to stimulate allogeneic T cell proliferation (***Fig. 4***), indicating that monocytes had differentiated into DCs in this culture system.

**Fig. 5:**
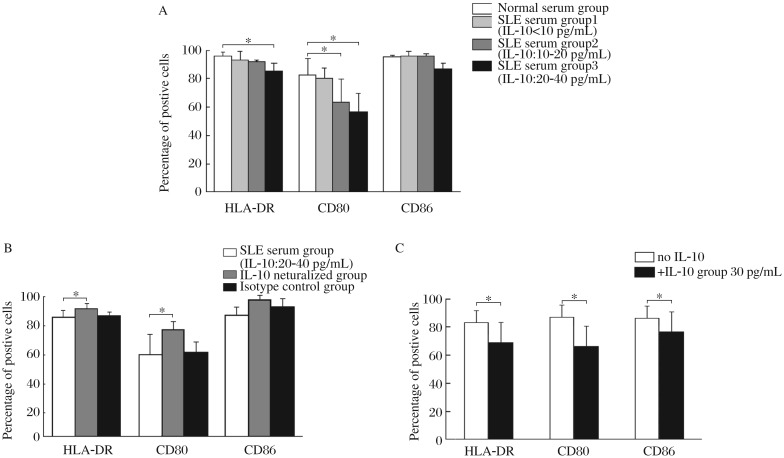
Effects of IL-10 on the phenotype of MDDCs induced by the GM-CSF + IL-4 + TNF-α culture system. A: MDDCs were induced by GM-CSF + IL-4 + TNF-α with normal serum alone or with SLE serum containing different levels of IL-10. SLE serum group 1, SLE serum with normal levels of IL-10 (<10 pg/mL); SLE serum group 2, SLE serum with mildly elevated levels of IL-10 (10-20 pg/mL); SLE serum group 3, SLE serum with highly elevated levels of IL-10 (20-40 pg/mL). B: MDDCs are induced by the GM-CSF + IL-4 + TNF-α culture system with SLE serum containing highly elevated levels of IL-10 (20-40 pg/mL) or SLE serum plus anti-human IL-10 neutralizing antibodies. SLE serum plus rabbit anti-human IgG is served as isotype controls. C: MDDCs are induced by GM-CSF + IL-4 + TNF-α with or without exogenous IL-10 (30 pg/mL). The expression of HLA-DR, CD80 and CD86 is determined by flow cytometry. Results are expressed as mean±SD of the percentage of positive cells. Data are obtained from six independent experiments. * *P* < 0.05.

In the GM-CSF + IL-4 + TNF-α culture system, MDDCs induced by SLE serum containing highly elevated levels of IL-10, expressed significantly reduced levels of HLA-DR and CD80 compared with those induced by the normal serum alone. The expression of CD80 on MDDCs induced by the SLE serum, containing mildly elevated levels of IL-10 also decreased (***Fig. 5A***). The inhibitory effects of SLE serum on the expression of HLA-DR and CD80 could be reversed by the addition of anti-IL-10 neutralizing antibodies into the SLE serum (***Fig. 5B***). Moreover, addition of exogenous IL-10 into the culture system resulted in decreased expression of HLA-DR, CD80 and CD86 by MDDCs compared with the normal controls (***Fig. 5C***).

**Fig. 6:**
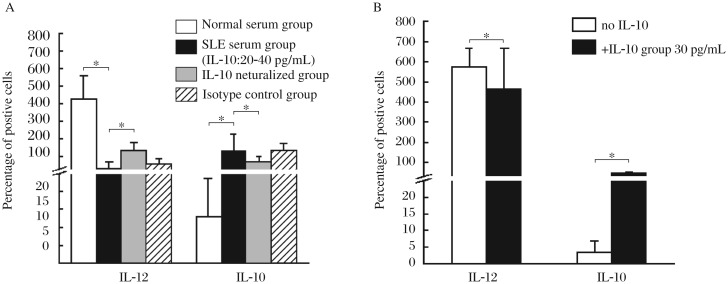
Effects of IL-10 on the cytokine production by MDDCs induced by the GM-CSF + IL-4 + TNF-α culture system. A: MDDCs are induced by GM-CSF + IL-4 + TNF-α with normal serum, SLE serum containing highly elevated levels IL-10 (20-40 pg/mL), SLE serum plus anti-human IL-10 neutralizing antibodies or SLE serum plus rabbit anti-human IgG isotype controls. B: MDDCs are induced by GM-CSF + IL-4 + TNF-α with or without exogenous IL-10 (30 pg/mL). The IL-12p40 and IL-10 concentrations in the culture supernatants of DCs are measured using ELISA kits. Results are expressed as mean±SD. Data are obtained from six independent experiments. * *P* < 0.05.

**Fig. 7:**
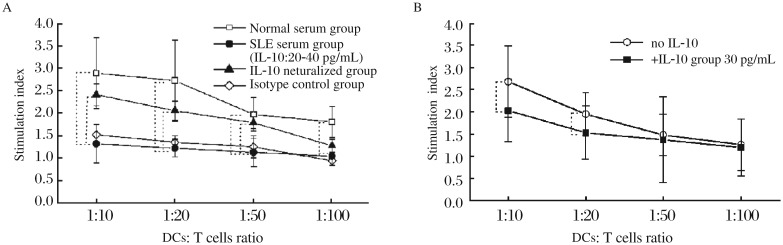
Effects of IL-10 on the allostimulatory capacity of MDDCs induced by the GM-CSF + IL-4 + TNF-α culture system. A: MDDCs are induced by GM-CSF + IL-4 + TNF-α with normal serum, SLE serum containing highly elevated levels IL-10 (20-40 pg/mL), SLE serum plus anti-human IL-10 neutralizing antibodies or SLE serum plus rabbit anti-human IgG isotype controls. B: MDDCs are induced by GM-CSF + IL-4 + TNF-α with or without exogenous IL-10 (30 pg/mL). DCs are then used as stimulator cells and incubated with allogeneic CD3^+^ T cells at DC:T cell ratios of 1:10, 1:20, 1:50 and 1:100. T-cell proliferation is measured using cell counting kit-8. The degree of proliferation is indicated as the stimulation index (SI). Results are expressed as mean±SD. Data are obtained from six independent experiments. * *P* < 0.05.

### IL-10 in SLE serum alters the cytokine production by MDDCs induced by the GM-CSF + IL-4 + TNF-α culture system

MDDCs induced by SLE serum containing highly elevated levels of IL-10 showed decreased production of IL-12p40, but increased IL-10 production, in comparison with those induced by the normal serum alone. Neutralization of IL-10 in the SLE serum reversed these changes (***Fig. 6A***). Addition of exogenous IL-10 into the culture system decreased the production of IL-12p40 and increased the secretion of IL-10 by MDDCs as compared with the normal controls (***Fig. 6B***).

### IL-10 in SLE serum decreases the allostimulatory capacity of MDDCs induced by the GM-CSF + IL-4 + TNF-α culture system

MDDCs induced by SLE serum containing highly elevated levels of IL-10 exhibited a reduced ability to stimulate allogenic T cell proliferation compared with those induced by normal serum alone at all DC:T cell ratios tested, and addition of anti-IL-10 neutralizing antibodies into the SLE serum reversed these changes at DC:T cell ratios of 1:10, 1:20 and 1:50 (***Fig. 7A***). Moreover, addition of exogenous IL-10 into the culture system decreased the allostimulatory capacity of MDDCs at DC:T cell ratios of 1:10 and 1:20 compared with the normal controls (***Fig. 7B***).

## DISCUSSION

Although SLE has been recognized primarily as a B-cell-induced disease characterized by the production of pathogenic auto-antibodies, the contribution of T cells to the activation of autoreactive B cells is unquestionable. It is well known that the activation of autoreactive B cells is dependent upon the assistance offered by T cells[22],[23], while the activation of autoreactive T cells is dependent upon stimulation by DCs. It has been demonstrated that IFN-α present in SLE serum induces monocytes to differentiate into DCs that are able to present self-antigens to T cells and activate autoreactive T cells[3], and both IFN-α and DCs have been considered to play central roles in the development of SLE[4]-[6],[24],[25]. Furthermore, IFN-α has been considered as a target for SLE treatment[26],[27]. However, IFN-α is not the only cytokine found to be abnormally elevated in the serum of SLE patients. Increased levels of IL-10 are seen in the serum of SLE patients more frequently than IFN-α. It has been shown that serum IL-10 levels and the number of IL-10-producing PBMCs or T lymphocytes are increased in SLE patients, and that IL-10 production is positively correlated with the production of anti-double stranded DNA (dsDNA) antibodies[28]-[30]. IL-10 has been shown to significantly affect the differentiation, maturation and function of DCs derived from monocytes. IL-10 inhibits the expression of HLA-DR, CD80, CD83 and CD86, and the production of IL-12 promotes the production of autocrine IL-10 and suppresses the allogenic T-cell stimulatory capacity of DCs[16]-[18],[31]. Whether IL-10 in the serum of SLE patients plays a similar role in the pathogenesis of SLE remains unknown. It was found that DCs from SLE patients expressed lower levels of HLA-DR, CD86 and CD9 than those from the normal controls[32]. In addition, there is a different response to IL-10 treatment between DCs derived from the monocytes isolated from SLE patients and those from normal controls. However, the abnormal effects of serum environment, particularly the increased level of IL-10 on the development and function of DCs in SLE patients are not well known.

In this study, the phenotypic and functional analyses were performed to investigate the effects of IL-10 present in the serum of SLE patients on the differentiation of DCs derived from monocytes. There are various methods for inducing monocytes to differentiate into DCs. One common method involves culture of monocytes with GM-CSF and IL-4. In 1998, a new method was established to induce human monocytes to differentiate into DCs using a transendothelial trafficking model[20]. In this model, monocytes migrate across an endothelial monolayer into the sub-endothelial collagen. The cells then reverse-transmigrate back to the apical surface of the endothelial monolayer. During this process, monocytes differentiate into DCs. This process, to a certain degree, simulates the in vivo differentiation of DCs from monocytes[33]. Then, the DC-differentiating potential of mouse monocytes was demonstrated using this transendothelial trafficking model[34].

The present study established a transendothelial trafficking model, and used it to induce human monocytes to differentiate into DCs. The results showed that, after incubation with an endothelial monolayer for 2 h, the proportion of CD11c^+^ and CD14^+^ cells in the PBMC population was reduced, indicating that monocytes had migrated through the endothelial monolayer into the sub-endothelial collagen. After the non-migrating cells were removed, the cells obtained after reverse transmigration displayed typical morphological and phenotypic characteristics, and immunostimulatory activity of DCs. In this transendothelial trafficking model, neutralization of IL-10 in the SLE serum by anti-IL-10 antibody increased the allostimulatory capacity of MDDCs, and addition of exogenous IL-10 into the normal serum decreased the allostimulatory capability of MDDCs. These results suggest that IL-10 in SLE serum has inhibitory effects on the allostimulatory capacity of MDDCs. Moreover, if SLE serum containing highly elevated levels of IL-10 was added into the culture system, the induced MDDCs showed reduced expression of CD80. Normal serum supplemented with exogenous IL-10 had similar effects, indicating that IL-10 in SLE serum inhibited the phenotypic maturation of MDDCs.

In order to further examine the effects of IL-10 in SLE serum, DCs were induced from monocytes using the GM-CSF + IL-4 + TNF-α culture system. After 8 d of culture, the cells showed high levels of HLA-DR, CD80, CD86, CD83 and CD1a, and a strong capacity to stimulate allogenic T-cell proliferation, as well as typical characteristics of DCs. After culture with GM-CSF + IL-4 + TNF-α, the MDDCs induced by the SLE serum showed reduced expression of HLA-DR (*P* < 0.05), CD80 (*P* < 0.05) and CD86 (*P* > 0.05) in an IL-10 dose-dependent manner compared with those induced by the normal serum alone. After neutralization of IL-10 in SLE serum, the expression of HLA-DR and CD80 was restored. These results suggest that the high levels of IL-10 present in SLE serum inhibit the expression of CD80 and HLA-DR by MDDCs. This finding is supported by the experiments incorporating exogenous IL-10. Furthermore, MDDCs induced by the SLE serum produced lower levels of IL-12p40 and higher levels of IL-10, and displayed a decreased capacity to stimulate allogenic T-cell proliferation compared with those induced by the normal serum alone. Neutralization of the IL-10 in SLE serum increased IL-12p40 production, decreased the production of IL-10 and increased the allostimulatory capacity of MDDCs. These results suggest that IL-10 present in the serum of SLE patients significantly affects the differentiation and function of MDDCs.

The mechanism underlying the inhibitory effects of IL-10 on MDDCs has been partially elucidated. It was found that the immunoregulation of IL-10 governing the maturation and activation of DCs was mediated via suppression of the phosphatidylinositol 3-kinase (PI3K)/Akt pathway and of Ikb kinase activity[35]. Recently, the mitogen-activated protein kinase (MAPK) signaling pathway was shown to play an important role in the maturation of DCs[36]. It has been known that IL-10 inhibits the surface co-stimulatory molecule expression and the stimulatory activity of human MDDCs by inhibiting the MAPKs, extracellular signal-regulated kinase 2 (ERK2), stress-activated protein kinase (SAPK)/c-Jun N-terminal kinase (c-JNK) and p38 MAPK pathways.

Actually, besides IL-10, abnormalities are found in a number of cytokines from patients with SLE[37]. IL-10 may modulate the development and function of DCs, as well as other cytokines and, thus, exhibit more complex effects. For example, it was reported that IFN-α could alter the function of IL-10 from an anti-inflammatory one to a proinflammatory one[38]. In IFN-α-primed and IL-10-stimulated cells, priming by IFN-α resulted in greatly enhanced signal transducers and activators of transcription1 (STAT1) activation in response to IL-10, subsequently induced IFN-γ-inducible protein-10 (IP-10/CXCL10) and finally induced IFN-γ and monokine production. Further studies should be carried out to investigate the complex effects of IL-10 combined with other cytokines.

In conclusion, our findings show that high levels of IL-10 in the serum of SLE patients significantly alters the phenotype, cytokine profile and the allostimulatory capacity of MDDCs, resulting in decreased expression of HLA-DR and CD80, reduced production of IL-12p40, increased production of IL-10 and a decreased capacity to stimulate allogenic T-cell proliferation. Alterations of the biological characteristics of DCs may result in defective T cell response to microbial antigens in SLE.

## Supplementary Figures

Fig. 1 Morphological identification of human umbilical vein endothelial cells (HUVECs). Freshly isolated HUVECs were spun onto slides and incubated with a rabbit antibody to human factor VIII-related antigen and a goat anti-rabbit IgG-FITC secondary antibody. Morphological analysis (A) was performed under fluorescence microscopy (original magnification×2000). HUVECs were then grown on polymerized collagen gels and cultured in RPMI 1640 supplemented with 20% FCS. By d 2, a confluent endothelial monolayer (B) was observed under an inverted phase-contrast microscope (original magnification: ×100).

Fig. 2 Morphological analysis of the reverse-transmigrated cells induced in the transendothelial trafficking model. A: Cells were spun onto slides, stained with Wright-Giemsa solution and observed by light microscopy (original magnification: ×100). B: Cells were fixed, dehydrated, dried, gilded, and analyzed by scanning electron microscopy (original magnification: ×8000).
