# Supplementary figures and images for: Serum IL-10 from systemic lupus erythematosus patients suppresses the differentiation and function of monocyte-derived dendritic cells
Source: J Biomed Res. 2012 Oct 31;26(6):456–66. doi: 10.7555/JBR.26.20120115 (PMC3597043; doi:10.7555/JBR.26.20120115)

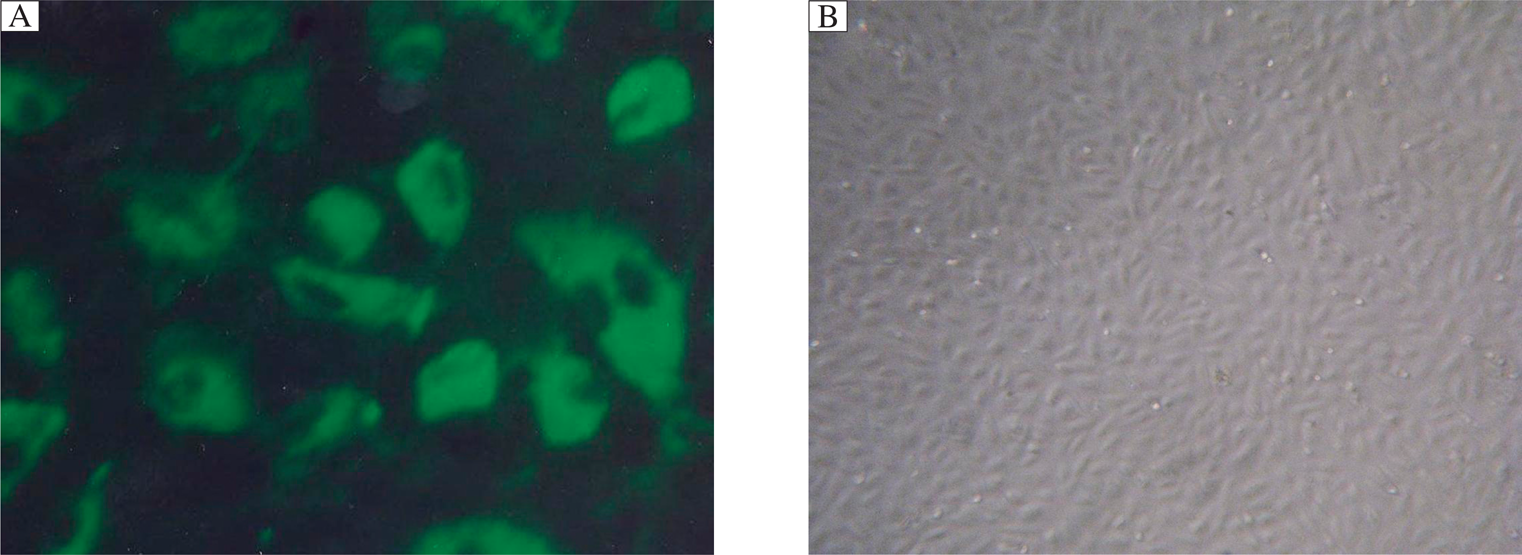

Supplement: Fig. 1 — Freshly isolated HUVECs were spun onto slides and incubated with a rabbit antibody to human factor VIII-related antigen and a goat anti-rabbit IgG-FITC secondary antibody. Morphological analysis (A) was performed under fluorescence microscopy (original magnification×2000). HUVECs were then grown on polymerized collagen gels and cultured in RPMI 1640 supplemented with 20% FCS. By d 2, a confluent endothelial monolayer (B) was observed under an inverted phase-contrast microscope (original magnification: ×100). [file jbr-26-06-456-s001.tif]

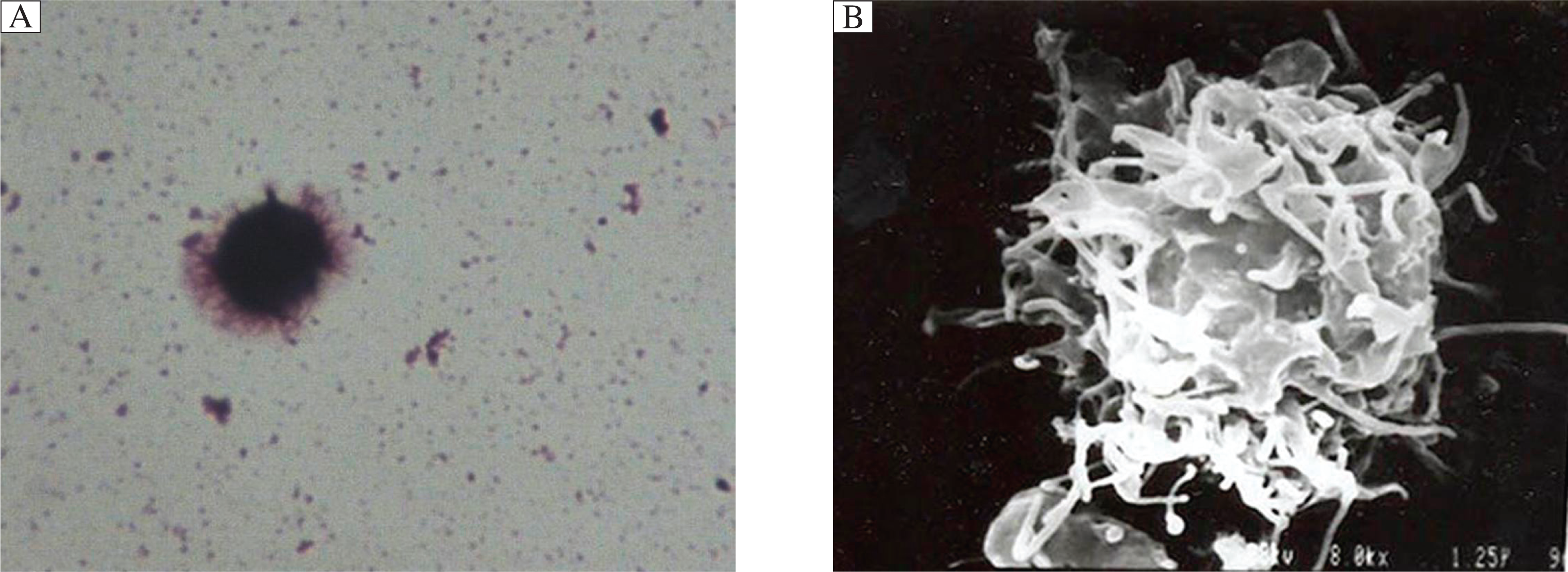

Supplement: Fig. 2 — A: Cells were spun onto slides, stained with Wright-Giemsa solution and observed by light microscopy (original magnification: ×100). B: Cells were fixed, dehydrated, dried, gilded, and analyzed by scanning electron microscopy (original magnification: ×8000). [file jbr-26-06-456-s002.tif]
